# Pharmacogenetics in critical care: association between *CYP3A5* rs776746 A/G genotype and acetaminophen response in sepsis and septic shock

**DOI:** 10.1186/s12871-023-02018-y

**Published:** 2023-02-16

**Authors:** C. Scorcella, R. Domizi, S. Amoroso, A. Carsetti, E. Casarotta, P. Castaldo, C. D’angelo, E. Damiani, F. Gasparri, A. Donati, E. Adrario

**Affiliations:** 1 Anesthesia and Intensive Care Unit, Azienda Ospedaliero Universitaria delle Marche, via Conca 71, Torrette di Ancona, 60126 Italy; 2 Department of Biomedical Sciences and Public Health, Università Politecnica Delle Marche, via Tronto 10/a, Torrette di Ancona, 60020 Italy

**Keywords:** Sepsis, Septic shock, Acetaminophen, Paracetamol, Personalization, Pharmacogenetic, Pharmacogenomic

## Abstract

**Background:**

Pharmacogenetics could represent a further resource to understand the interindividual heterogeneity of response of the host to sepsis and to provide a personalized approach to the critical care patient.

**Methods:**

Secondary analysis of data from the prospective observational study NCT02750163, in 50 adult septic and septic shock patients treated with Acetaminophen (ACT) for pyrexia. We investigated the presence of two polymorphisms, located respectively in the genes *UGT1A1* and *CYP3A5,* that encode for proteins related to the hepatic metabolism of ACT. The main dependent variables explored were plasmatic concentration of ACT, body temperature and hepatic parameters.

**Results:**

8% of the patients carried *CYP3A5* rs776746 A/G genotypes and showed significantly higher plasma levels of ACT than GG wild type patients, and than patients with *UGT1A1* rs8330 C/G genotypes.

**Conclusions:**

Identifying specific genotypes of response to ACT may be helpful to guide a more personalized titration of therapy in sepsis and septic shock. *CYP3A5* might be a good biomarker for ACT metabolism; however further studies are needed to confirm this result.

**Trial registration:**

NCT02750163.

## Background

The concept of “personalizing the therapeutic approach to the individual” is gaining crescent interest, in particular when applied in critical care setting [1–3]. Personalizing the approach means to analyze multiple aspects of both the disease and the patient and to identify the most targeted management for the single scenario.

Pharmacogenetics (PGx) is a relatively young research field; it aims to translate laboratory genetic test information into clinical practice to help to understand inter-individual differences among patients and develop more accurate drug dosing [4–6].

There are pharmacogenetic clinical guidelines, as the Clinical Pharmacogenetics Implementation Consortium (CPIC) and the The Dutch Pharmacogenetics Working Group (DPWG) guidelines, that provide pharmacogenetics-based dose-adjustment recommendations for drugs with strong evidence of a correlation between genetics and clinical response in terms of pharmacokinetics and pharmacodynamics, but further studies are needed to extend our knowledge in this field and use it routinely in clinical practice [7, 8].

So far, PGx has been investigated mainly in the area of subacute and chronic illness (cardiovascular diseases, cancer, immunological disorders), little attention has been paid to the potential of pharmacogenetics in the critical care environment, although the most commonly used medications have been already explored [4].

Acetaminophen (ACT) is one of the most common antipyrexial molecules used for the treatment of high body temperature in sepsis, but the response in terms of defervescence is heterogeneous in the population. To date, there is limited evidence about the role of PGx in the inter-individual response to ACT.

Between July 2017 and 2019 we performed a prospective observational study to evaluate the effect of ACT on the sublingual microcirculation of pyrexial septic patients (NCT02750163, first registration 25/04/2016). We aimed to investigate, as secondary outcome of the study, what was the prevalence in our sample of *CYP3A5* rs776746 and *UGT1A1* rs8330 polymorphisms, and if a correlation existed between the presence of any of the two polymorphisms and ACT plasmatic concentration and body temperature reduction [6, 9].

*CYP3A5* is the gene that encode for cytochromeP450 (CYP) 3A5 that is a protein responsible for converting ACT to N-acetylparaquinoneimine (NAPQI, an hepatotoxic metabolite) that is then detoxified by conjugation with glutathione. Rs776746 identify the genetic variant (A/G, at the genomic position 22,893 on chromosome 7) of *CYP3A5* that is responsible for aberrant splicing of *CYP3A5* mRNA, that results in a nonfunctional protein [6, 9].

UDP-glucuronosyltransferase (UGT1A1) rs8330 is a single nucleotide C/G polymorphism in the *UGT1A* gene, associated with increased hepatic ACT clearance through glucuronidation (probably through effects on *UGT1A* gene splicing). In 2013 Courth et al. studied polymorphisms in genes encoding the acetaminophen UGT enzymes, to try to explain the interindividual variability in risk for liver injury after acetaminophen overdose. They showed that the glucuronidation activity of ACT in rs8330 C/G heterozygotes was significantly higher than in wild types C/C homozygotes and it was also significantly lower than the variant rs8330 GG homozygotes [9, 10].

These data suggest that further research could be of interest to understand if a specific pharmacokinetic screening of these genotypes could help to personalize the treatment with ACT in critical care patients.

## Methods

NCT02750163 is a prospective observational study conducted in the University Intensive Care Unit (ICU) of Azienda Ospedaliero Universitaria delle Marche. It included 50 adult patients with sepsis or septic shock and high body temperature, treated with ACT on clinical judgment. Patients that received ACT in the previous 12 h were excluded. Before the administration of ACT (t0), 30 min after the end of the infusion (t1) and 2 h later (t2), plasma concentration of ACT and central body temperature were measured as part of the clinical parameters investigated for NCT02750163 that included hemodynamic and respiratory parameters, lactate, blood gas samples, diuresis and fluid balance. Liver function was explored at t0.

*UGT1A1* rs8330 (NM_000463.3:c.*440G > C) and *CYP3A5* rs776746 (NG_007938.2:g.12083A > G) were analysed from wool blood samples at t0, with QIAamp DNA Mini and Blood Mini (Qiagen) kit for DNA extraction and Nanodrop quantification, assayed by rt-PCR (reverse transcription-Polymerase Chain Reaction) method.

Concentration of ACT was measured on plasma with the homogeneous enzyme immunoassay DRI® Acetaminophen Serum Tox Assay (Thermo Scientific).

This sub-analysis was performed using Graphpad Prism 6 and Microsoft Excel 16. According to the distribution of the main variables (assessed with Kolmogorov–Smirnov test) data are presented as medians and Interquartile Ranges (IqR), or numbers and percentages.

Non-parametric Mann–Whitney U was used to compare the differences in body temperature reduction and plasmatic concentration according to the genotypes *p* < 0.05 for significance.

In compliance with national applicable laws, informed consent was obtained from the subjects before inclusion; patients that were temporarily unable to consent for neurological impairment or sedation were included in the study with deferred subject consent and written informative for the next of kin at the time of enrollment. The study protocol was approved by the Local Ethics Committee (Comitato Etico Regione Marche—CERM) and it conformed to the principles of Helsinki declaration.

In this brief report we describe the characteristics of our sample and the results of the analysis.

## Results

Of the 50 patients, 80% were males, with a mean age of 58 (± 16) years. Sepsis derived mainly from lower respiratory and genitourinary tract infections (respectively 56% and 20% of the total). Median SOFA score at entrance in ICU was 10 [6–10].

All the patients received a dose of 1 g of ACT in 30 min of infusion with a median of 11.11 mg/kg [10.00–12.71] of ACT administered. The plasma concentration of ACT evaluated at t0, t1 and t2 was 1.04 mcg/ml [0.00–2.02], 11.85 mcg/ml [9.30–16.20], 7.55 mcg/ml [5.05–8.42]. In 27 patients (54%) the concentration of ACT at t1 was in range (normal range 10–20 mcg/ml), while it was under range in 17 patients (34%, 8.30 [7.30–9.40] mcg/ml) and over-range in 6 patients (12%, 23.69 [20.80–31.10] mcg/ml). The median body temperature was 38.3 °C [38.1–38.8] at t0, 38.1 °C [37.9–38.6] at t1, 37.9 °C [37.5–38.3] at t2. There was no difference in defervescence between patients with normal, over or under-range levels of ACT.

Thirteen patients (26%) carried *UGT1A1* rs8330 C/G genotype, 4 patients (8%) carried *CYP3A5* rs776746 A/G genotype. No UGT1A1 rs8330 G/G or CYP3A5 rs776746 A/A patients were found.

**Fig. 1:**
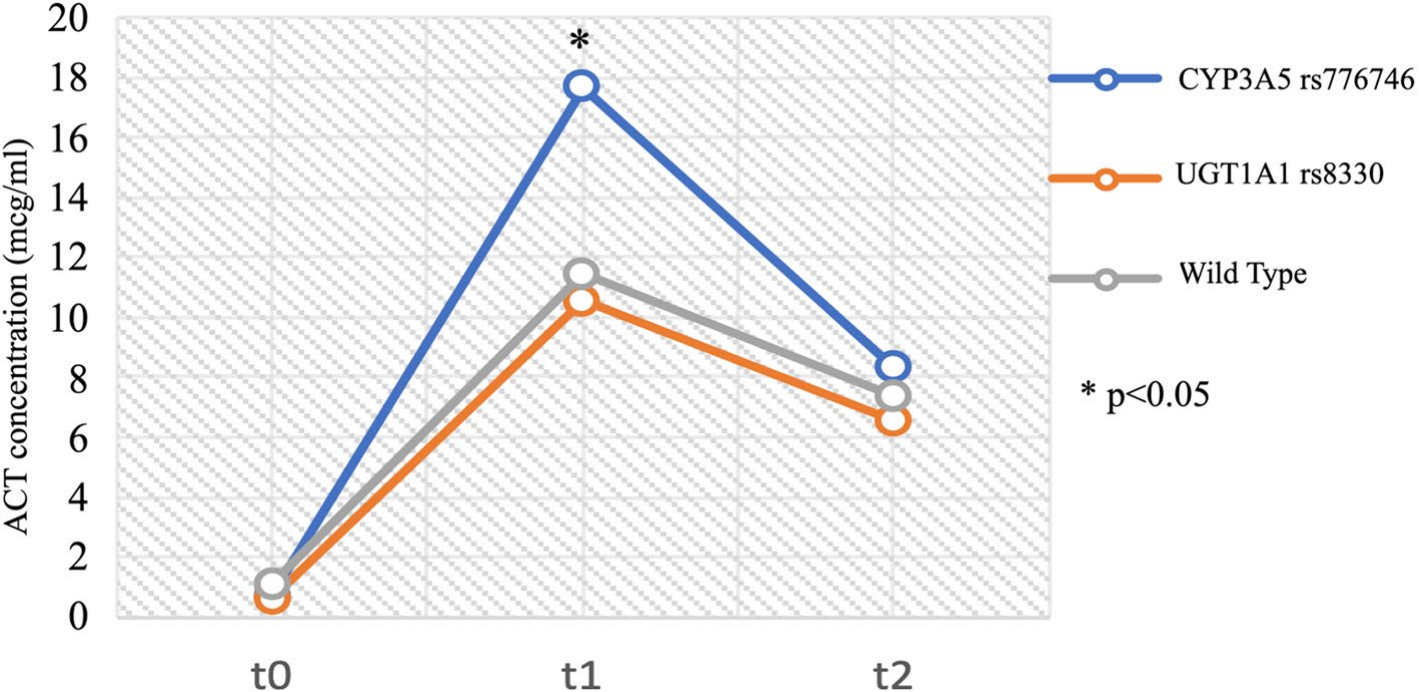
Plasma concentration of ACT in WT, in patients with *CYP3A5* rs776746 A/G and *UGT1A1* rs8330 C/G genotypes at t0 (before the administration of ACT), t1 (thirty minutes after the infusion) and t2 (two hours after the infusion). *p < 0.05 at t1, Mann Whitney U test for *CYP3A5* rs776746 A/G versus WT and *CYP3A5* rs776746 A/G versus *UGT1A1* rs8330 C/G genotype

At t1, patients that carried *CYP3A5* rs776746 A/G genotype had higher plasma concentration of ACT (17.60 mcg/ml [16.20–21.69]) than G/G patients (11.4 mcg/ml [9.10–14.29]; *p* = 0.03), and than patients with *UGT1A1* rs8330 C/G genotype (10.50 mcg/ml [9.30-13.10]; *p* = 0.02), Mann–Whitney U test (Fig. 1).

No differences in plasmatic concentration of ACT among genotypes were found at t2.

Liver function measured at t0 showed no difference between *CYP3A5* rs776746 A/G and *UGT1A1* and rs8330 C/G patients when compared to *CYP3A5* rs776746 G/G and *UGT1A1* rs8330 C/C patients (Table 1).

**Table 1:** Liver function tests measured at t0 in Wild Type patients, and patients with *CYP3A5* rs776746 A/G and *UGT1A1* rs8330 C/G genotypes. Median, IqR

	Wild type (WT)	*CYP3A5* rs776746 A/G genotype	*UGT1A1* rs8330 C/G genotype
Aspartate transaminase, IU/L	47.50 [32.75–97.25]	61.00 [48.00–61.00]	52.00 [30.75–83.75]
Alanine transaminase, IU/L	40.00 [22.25–99.00]	36.00 [25.00–36.00]	40.50 [27.50–71.75]
Lactate dehydrogenase, IU/L	285.50 [211.50–385.75]	252.00 [187.00–252.00]	248.50 [219.50–343.50]
International Normalized Ratio, AU	1.31 [1.22–1.40]	1.29 [1.26–1.34]	1.26 [1.20–1.48]
Bilirubin, mg/dL	0.80 [0.65–1.70]	1.30 [0.95–2.15]	1.35 [0.60–2.60]

The average dose of ACT administered per kg of body weight was not statistically different in *CYP3A5* rs776746 A/G and *UGT1A1* rs8330 C/G patients when compared to *CYP3A5* rs776746 G/G and *UGT1A1* rs8330 C/C.

**Fig. 2:**
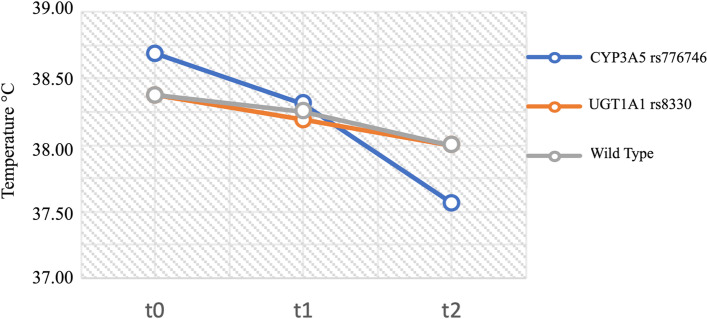
Body temperature of WT, *CYP3A5* rs776746 A/G and *UGT1A1* rs8330 C/G patients at t0 (before the administration of ACT), t1 (thirty minutes after the infusion) and t2 (two hours after the infusion). Mann–Whitney U test

The deltha temperature t2-t0 was more evident in *CYP3A5* rs776746 A/G patients compared to *CYP3A5* rs776746 G/G and to *UGT1A1* rs3880 C/G genotypes (∆T -0.85 °C [-1.65–0.02] versus -0.40 °C[-0.80–0.05] and -0.45 °C [0.65–0.10]), but the difference was not statistically significant (Fig. 2). The deltha temperature t1-t0 and t2-t1 were not statistically significant.

## Discussion

Acetaminophen is widely used in intensive care for both antipyretic and analgesic effects.

The extended inter-patient variability limits its efficacy and therapeutic index, and expose patients to drug toxicity and to therapeutic failure.

While pharmacogenetic approach have been applied for a variety of other drugs, studies addressing the role of genetic variability of ACT are still limited.

In this secondary analysis of the prospective observational study NCT02750163 we focused the attention on two polymorphisms of the metabolism of ACT in 50 critical care septic patients, and on their association with the levels achieved by ACT in plasma and with temperature reduction 30 min and 2 h after the administration of ACT.

A limited percentage of the patients included in the sample carried *CYP3A5* rs776746 A/G genotype, all of them in heterozygosis; of interest, the patients with this genotype showed significantly higher peaks of plasma concentration of ACT if confronted with the rest of the population and also a trend toward a higher defervescence.

This result is consistent with previous studies on the metabolism of ACT and with the information reported in the allele functionality table for *CYP3A5* (https://www.pharmgkb.org/haplotype/PA166128219), that defines the polymorphism rs776746 as a genotype characterized by a loss of function in the enzyme CYP450 3A5 [11–13]. As Cytochrome P450 3A5 is involved in the metabolization of ACT to NAPQI, these data can suggest that the individuals who carry *CYP3A5* rs776746 A/G genotype can show higher plasma level of ACT because of slower NAPQI transformation of the drug for lack of CYP3A5 activity (compared to individuals with *CYP3A5* rs776746 G/G genotype).

*UGT1A1* rs8330 heterozygous genotype was frequent in our sample, but it was not correlated with the plasma level of ACT achieved nor to a different pattern of body temperature reduction.

The hypothesis that guided the selection of this specific polymorphism was that patients that carry C/G polymorphism in the UDP-glucoronosyltransferase1 A could present lower ACT level because of increased hepatic ACT clearance. We suggest that the analysis of other *UGT1A1* polymorphisms (for example rs10929303, rs1042640) could be of interest for further research, as the impact of these polymorphisms on enzyme activity is better known [10, 14].

In our sample, the plasma level of ACT was lower than in patients with *CYP3A5* rs776746 genotype but similar to the wild-type population. As the polymorphism was present only in heterozygosis, it could be of interest to understand if individuals with the same polymorphism present in homozygous could behave to ACT differently.

The mechanism that explains this association need further studies, however both the polymorphisms that we investigated were reported from the Acute Liver Failure Study Group as genetic forms that are linked with predisposition to acetaminophen-induced acute liver failure, either in intentional overdose of ACT and in unintentional chronic over-use of the drug [9, 10].

There are important limitations to this study: this is a secondary analysis on a small population of patients where the presence of the two polymorphisms, in particular of *CYP3A5* rs776746 was limited to a restrained percentage of cases; the study was not designed nor powered to answer a cause-effect question or to perform more solid statistical analysis, therefore the results cannot be considered conclusive in any sense. Moreover, the polymorphisms were carried in heterozygosis and none of the patients presented both the polymorphisms, therefore further studies should investigate whenever the presence of the polymorphism in homozygosis or the occurrence of the two polymorphisms in the same individual may have a different impact on the associations we found.

On the other end, the important message that the results give is that PGX has not been adequately investigated in the context of critical care; our data should be read with caution, but we believe they could be of interest to encourage future trials on the role of pharmacogenetics in the use of medications in critical care and in particular in sepsis and septic shock.

There are several aspects that influence the way the body handle with medications, including age, gender, concomitant drug therapy and the critical illness itself. Genetic profile, undoubtedly is one of them and its role in sepsis is under-estimated. Identifying specific polymorphisms may help to explain drug-response genotypes and the application of pharmacogenetic in the clinical scenario may help to guide the clinician to a more patient-titrated therapy in sepsis [15–18].

Several randomized controlled trials have already demonstrated clinical utility (on improving the outcome) of genotype-guided therapy versus usual care in several diseases, and several drug labels already include pharmacogenetic recommendations to be conducted routinely prior to drug administration (for example, *CYP2C9* and *VKORC1* genetic variation on warfarin dosing requirements and risks) [5, 8, 19, 20]. We suggest that a similar approach should be investigated also in the context sepsis and septic shock.

## Conclusions

This secondary analysis describes the prevalence of two polymorphisms of the metabolism of ACT is a small population of adult septic patients. It reports a significant association between *CYP3A5* rs776746 A/G genotype and ACT blood levels. In a future perspective, clinical implementation of PGX could show application in critical care, but further studies are needed to confirm these results.

## Data Availability

The data are available on reasonable request to roberta.domizi@ospedaliriuniti.marche.it.

## Abbreviations

ACT: Acetaminophen
ICU: Intensive Care Unit
PGx: Pharmacogenetics
SOFA: Sequential Organ Failure Assessment
